# Case report: Therapeutic drug monitoring and CYP2D6 phenoconversion in a protracted paroxetine intoxication

**DOI:** 10.3389/fphar.2024.1444857

**Published:** 2024-09-04

**Authors:** Alena Damborská, Lenka Hanáková, Eva Pindurová, Kateřina Horská

**Affiliations:** ^1^ Department of Psychiatry, University Hospital Brno and Faculty of Medicine, Masaryk University, Brno, Czechia; ^2^ CEITEC - Central European Institute of Technology, Masaryk University, Brno, Czechia; ^3^ Department of Psychiatry, University Hospital Brno, Brno, Czechia; ^4^ Center of Molecular Biology and Genetics, University Hospital Brno, Brno, Czechia; ^5^ Laboratory of Clinical Microbiology, Forlab Ltd., Brno, Czechia; ^6^ Department of Pharmacology and Toxicology, Faculty of Pharmacy, Masaryk University, Brno, Czechia; ^7^ Department of Clinical Pharmacy, Hospital Pharmacy, University Hospital Brno, Brno, Czechia

**Keywords:** paroxetine, poor and intermediate metabolizer, CYP2D6, phenoconversion, overdose, case report

## Abstract

**Objective:**

The cytochrome P450 2D6 (CYP2D6) is an enzyme involved in the oxidative biotransformation of various widely used drugs, including paroxetine, a substrate and strong inhibitor of the enzyme. The aim is to report on a case of protracted intoxication with paroxetine after a single overdose in a genotype-predicted intermediate CYP2D6 metabolizer.

**Observation:**

A 49-year-old man was receiving chronic treatment for more than 6 years with paroxetine 60 mg/day for an indication of agoraphobia. The patient ingested fifty 20 mg tablets of paroxetine in a suicide attempt. The toxic plasma level, accompanied by delirium, persisted for approximately 1 month after the overdose. According to the genotype profile, the patient was evaluated as an intermediate metabolizer with reduced CYP2D6 enzyme activity.

**Conclusion:**

As a consequence of the suicide attempt with overdose and the chronic paroxetine treatment that preceded it, phenoconversion to a poor metabolizer with very low CYP2D6 enzyme activity is suggested as contributing to an extremely long intoxication accompanied by delirium. Prolonged monitoring over a standard 24 h of both physical symptoms and drug plasma levels, together with a genetic profile assessment and phenoconversion consideration, is recommended after a single overdose in patients chronically treated with paroxetine.

## 1 Introduction

The cytochrome P450 2D6 (CYP2D6) is an enzyme involved in the oxidative biotransformation of various widely used drugs, including antidepressants such as selective serotonin reuptake inhibitors (SSRIs) (Sindrup et al., 1992; Bousman et al., 2023). This enzyme is encoded by a highly polymorphic CYP2D6 gene. A wide variability in the CYP2D6 gene is reflected in inter-individual and inter-ethnic differences in medication response (Ingelman-Sundberg, 2005). Substantial evidence supports a close relationship between the CYP2D6 genotype and phenotypic variability (Bousman et al., 2023) in pharmacokinetic parameters and treatment outcomes (Žourková and Hadašová, 2003; Hole et al., 2023).

To date, over 170 haplotypes (or star (*) alleles) have been defined by the Pharmacogene Variation (PharmVar) Consortium relevant to the CYP2D6 gene (Gaedigk et al., 2018). In interpreting the CYP2D6 individual genetic test, a metabolic phenotype is estimated as the sum of enzyme activity values, i.e., the functional capacity, of all reported CYP2D6 alleles. According to these genotype-predicted phenotypes, which vary from reduced to extremely high total enzyme activity, individuals are evaluated as follows: poor metabolizers (PMs), intermediate metabolizers (IMs), normal metabolizers (NMs), and ultrarapid metabolizers (Bertilsson et al., 2002; Bousman et al., 2023). The frequency of each phenotype differs across world populations and ethnic groups (Gaedigk et al., 2017; Vichi et al., 2021; Bousman et al., 2023). About 5%–10% of the Caucasian population carries non-functional alleles manifesting as IM or PM phenotypes (Bousman et al., 2023; Koopmans et al., 2021; Pelkonen et al., 2008). More than 90% of all non-functional alleles are CYP2D6*3 (2637delA, with A deletion in exon 5), CYP2D6*4 (1934G > A, with G to A mutation at the intron 3-exon 4 junction), and CYP2D6*5 (deletion of the entire CYP2D6 gene) (Sistonen et al., 2007).

However, the metabolic activity of the CYP2D6 enzyme is affected not exclusively by the genotype. Importantly, it depends on a wide range of intrinsic (patient- and disease-related) factors, e.g., sex, smoking, inflammation, and on various extrinsic factors, e.g., concomitant medication, food supplements, dietary and nutritional factors, xenobiotics, etc. This phenomenon, called phenoconversion, is defined as: “a mismatch between the individual’s genotype-based prediction of drug metabolism and the actual capacity to metabolize drugs” (Klomp et al., 2020).

Administration of drugs that are strong CYP2D6 inhibitors has a significant clinical impact, resulting in phenoconversion into the lower metabolic, or PM, status (Pelkonen et al., 2008; Žourková and Hadašová, 2003; Hakkola et al., 2020). In general, the relevant factors affecting drug-induced phenoconversion and consequent metabolic status involve not only the genotype and the inhibitory effects of the respective drug, e.g., the strength of inhibition, classification of the inhibitor as weak, moderate, or strong (Klomp et al., 2020), but also the dose of the inhibitor, the steady state condition (Bousman et al., 2023) and alternative biotransformation pathways of the substrate. However, in a clinical setting, the limited understanding of phenoconversion prevents precise predictions of the extent of enzyme isoform metabolic activity inhibition and impact on drug biotransformation in an individual patient. Importantly, this further underscores the benefits of the use of therapeutic drug monitoring (TDM) in clinical practice. TDM represents an effective strategy for personalized therapy using quantification and interpretation of plasma drug concentrations to optimize pharmacotherapy (Hiemke et al., 2018).

Paroxetine, one of the SSRIs, is both a substrate and a strong inhibitor of the CYP2D6 enzyme. Dosing recommendations on antidepressants, including paroxetine, were formulated recently that take into account the aspect of phenoconversion (Bousman et al., 2023). Here, we report a case in which we observed a clinical picture of delirium with protracted intoxication for approximately 1 month after a suicide attempt by swallowing a 1 g overdose of paroxetine in a patient who was chronically treated with paroxetine 60 mg/day and genotyped as IM. Paroxetine-induced phenoconversion to PM is suggested to have contributed to the protracted intoxication in this patient.

## 2 Case report

### 2.1 Medical history

A 49-year-old Caucasian man with a 26-year history of agoraphobia with panic disorder was admitted to the Department of Internal Medicine in February 2021 a few hours after a suicide attempt consuming around 50 20 mg tablets of paroxetine (Apo-Parox^®^), i.e., 1 g of paroxetine. Before the suicide attempt, he had been treated with paroxetine 60 mg/day for more than 6 years. Thus, the overdose represented a 17-times higher dose than the patient’s chronic daily dose. Before this hospitalization, the patient was a smoker (20 cigarettes per day), he had a 10-year history of benzodiazepine abuse, he had no history of alcohol abuse or any other substance abuse. Apart from hypertension, for which he was intermittently taking metoprolol, he had not been treated for any medical conditions. The medication regimen prior to the overdose was as follows: clonazepam 6 mg/day, alprazolam 2 mg/day, paroxetine 60 g/day. No other concomitant medication was prescribed; neither the patient nor his family reported the use of any over-the-counter drugs or dietary supplements, and so any drug interactions with clinical relevance to paroxetine pharmacokinetics or effects were not present before admission.

Prior to the hospitalization, according to the patient’s wife, the patient stopped going out and was more anxious in relation to the COVID-19 pandemic. During the 2 weeks before the suicide attempt, he was very dependent on her. Nevertheless, to her knowledge, he was not speaking incoherently, hallucinating, or communicating suicidal thoughts or tendencies.

### 2.2 Initial medical examination and treatment

**FIGURE 1:**
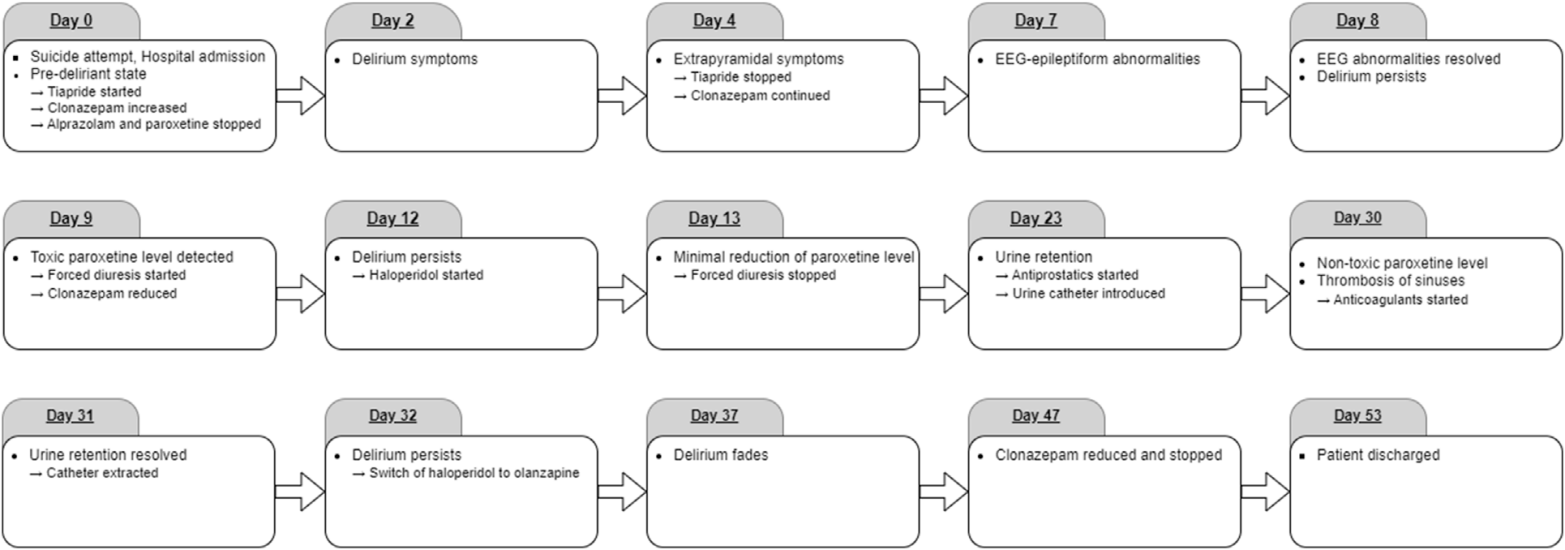
Timeline of the events described.

On the day of admission to the hospital (day 0, see Figure 1), the patient presented symptoms such as fluctuating lucidity of consciousness, psychomotor restlessness, severe anxiety with tremor, emotional lability, intrapsychic tension, subdepressive mood, hyperhidrosis, and constipation. Moreover, the patient presented auto-aggressive behavior such as stabbing himself in the neck with a pencil and putting a toothbrush in his eye. The patient’s appearance was badly neglected, with dental caries, defective teeth, overgrown nails, sore spots in the groin, and hyperkeratotic foci on the elbows and knees. He had poor oral intake of food and fluids due to his social condition and lack of adequate resources. Liver function tests, including liver transaminases, alkaline phosphatase, gamma-glutamyl transferase, and serum bilirubin, were performed on the day of admission and then regularly during the whole hospitalization, and no pathology was found. Routine imaging tests, including abdominal ultrasound, chest radiography, and brain-computer tomography, revealed no abnormalities. Gastric lavage was not indicated. Routine laboratory tests showed increased levels of C-reactive protein (CRP, 47 mg/L), and urine screenings were positive for benzodiazepines. Cefuroxime was administered to empirically treat a suspected urinary infection, followed by a decrease in CRP levels over the next few days. Following a liaison with psychiatric specialists, a pre-deliriant state and withdrawal symptoms within the benzodiazepine addiction were suspected based on the patient’s clinical status and medical history of benzodiazepine abuse. Therefore, after a standard 24-h vital signs monitoring, the patient was transferred to the local Department of Psychiatry. At this time, the patient was treated with an atypical antipsychotic tiapride 200 mg/day and clonazepam 7.5 mg/day intravenously, although tiapride was discontinued on day 4 due to extrapyramidal symptomatology. Paroxetine was no longer administered after intoxication and admission to the hospital.

### 2.3 Standard psychiatric care during the first week after intoxication

On admission to the Department of Psychiatry on day 2, delirium symptoms dominated the clinical picture, including disorientation, restlessness, and emotional lability. The patient’s speech was incoherent “word salad.” Because of agitation, intermittent physical restraint was necessary.

Electroencephalography (EEG) on day 7 after the intoxication revealed a generalized nonspecific abnormality and a specific epileptiform abnormality with the right fronto-temporo-central maximum. There was little clinical improvement at that time; therefore, to further investigate the cause of the persisting delirium, the patient was transferred on day 8 to a higher level of care department, the intensive care unit of the Department of Psychiatry, Medical Faculty of Masaryk University and University Hospital Brno.

### 2.4 Intensive psychiatric care during the days 8 and 9

On admission, the patient was alert, completely disoriented, and producing non-cooperative, emotionally incontinent behavior with active negativism, verbigeration, and grimacing. No aggressive behavior was observed. Standard laboratory testing, abdominal ultrasound, and chest radiography revealed no pathology. Repeated EEG returned normal results; the abnormalities seen earlier were now resolved.

**FIGURE 2:**
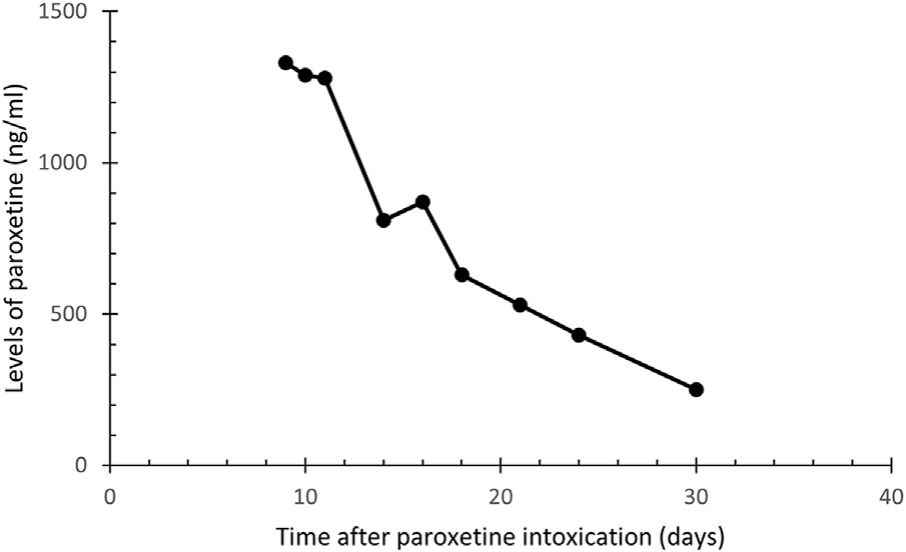
Plasma concentration of paroxetine in a patient after paroxetine intoxication.

On day 9 after the intoxication, the first available result of paroxetine plasma level was 1,330 ng/mL, i.e., more than three times the level considered as toxic 350 ng/mL (Hiemke et al., 2018; Schulz et al., 2020). This corresponds to the half-life (t1/2) of approximately 9 days; the calculation is based on the concentration curve (see Figure 2). Since no specific antidote for paroxetine poisoning exists, forced diuresis with furosemide 20 mg/day and spironolactone 200 mg/day was commenced immediately. Additionally, intravenous administration of clonazepam was now reduced to 6 mg/day.

### 2.5 Intensive cardiologic care

On day 10, tremors of the trunk, athetoid movements, hypertension, tachycardia, and fever were observed, and therefore, epileptic seizure was suspected. Nevertheless, a neurologic examination showed no lateralization, and no focal or epileptiform EEG abnormalities were observed during repeated testing in the following weeks of hospitalization. Given the low myoglobin and creatine kinase levels and no exposure to antipsychotics, neuroleptic malignant syndrome was unlikely according to diagnostic criteria (Gurrera et al., 2017), and symptoms of serotonin syndrome (Scotton et al., 2019) were not fully manifested. Nevertheless, due to persistent toxic levels of paroxetine and persistent delirium symptoms accompanied by hypertension, tachycardia, and fever, the patient was transferred to the intensive care unit of the internal cardiology clinic.

Besides the sinus tachycardia, however, no malignant arrhythmia was observed during a 24-h monitoring of cardiac parameters, and the echocardiography was also without any cardiac pathology. Therefore, the patient was transferred back to the intensive care unit of the Department of Psychiatry.

### 2.6 Further intensive psychiatric care 2 weeks after intoxication

Since the levels of paroxetine decreased only minimally during 5 days of forced diuresis, the forced diuresis was ended on day 13, and only symptomatic treatment was continued. Psychotic symptomatology dominated the clinical picture, including commenting auditory hallucinations, verbigeration, grimacing, and suicidal proclamations without tendencies to realization. To suppress these symptoms, administration of haloperidol was commenced on day 12 and titrated up to 10.5 mg/day. Nevertheless, the psychotic symptomatology persisted, including religious delusions, so haloperidol was switched to olanzapine on day 32 and set to 15 mg/day with monitoring of plasma concentrations. Paroxetine levels were monitored (see Figure 2.) and slowly dropped to 250 ng/mL on day 30, thus reaching the level considered non-toxic not sooner than 1 month after the intoxication. Clonazepam was switched to oral administration from day 16, and the dose was gradually decreased and completely discontinued on day 47.

### 2.7 Somatic complications and comorbidities

Due to urine retention, a permanent urine catheter was introduced, administration of antiprostatic treatment with tamsulosin hydrochloride 0.4 mg/day commenced on day 23, and the catheter was successfully extracted after 1 month on day 31. The urine retention was considered drug-induced, associated with prominent anticholinergic paroxetine effects. Thus, anticholinergic delirium was also assumed, though no other typical symptoms, such as dry mucosa and skin, were present. In the clinical status dominated increased serotonergic activity, e.g., excessive perspiration. Benzodiazepines were administered to prevent epileptic seizure and full manifestation of serotonergic toxicity.

To rule out any structural cause of the persistent delirium, magnetic resonance imaging of the head was performed on day 30; it showed thrombosis of the left sigmoid and transverse sinuses. Although subsequent lab testing did not reveal any cause of the hypercoagulable state, full anticoagulant treatment was commenced with low molecular weight heparin; this was later changed to non-vitamin K oral anticoagulant dabigatran etexilate 300 mg/day.

### 2.8 Genotype-based phenotype prediction

To shed light on underlying inborn predispositions to persisting high plasma levels of paroxetine, the patient’s genetic profile was made on day 31. Molecular genetic polymerase chain reaction-based analysis of the CYP2D6 gene was performed using the LightMix Kit to detect single nucleotide variants (the allele *3, represented by the core polymorphism 2549 delA (rs35742686) and the allele *4, represented by the core polymorphism 1846G > A (rs3892097)), and a copy number variation (CNV), particularly a complete deletion of the CYP2D6 gene (allele *5). While maintaining the economic feasibility of the examination, these three alleles (*3, *4 and *5) were chosen based on their high prevalence in the Caucasian population and their known impact on CYP2D6 enzyme activity as non-functional alleles. Additionally, specific primers were utilized to test for presence of other relevant CNVs, which substantially impact CYP2D6 enzyme activity: duplications of the entire CYP2D6 gene and duplications of alleles *1 and *2. Alleles with reduced function, e.g., 9*, *10, *17, *36, *41, or other less frequent non-functional alleles (Raimundo et al., 2000) were not tested. Non-functional alleles can be caused by point mutations (substitutions, deletions, insertions), which disrupt the reading frame, interfere with proper splicing, or lead to premature termination of translation (e.g., alleles *6, *8, *11, *15, *19, *20, *38, *40, *42, *44), by large chromosomal deletions (alleles *13, *16) or by mutations leading to loss of protein function while its length remains preserved (e.g., alleles *7, *12, *14, *18) (Bousman, et al., 2023).

In the Caucasian population, the most common non-functional allele is *4, with a frequency of around 20%. The *5 allele occurs in all populations with a frequency of 2%–7% (Raimundo et al., 2000) and the*3 allele’s frequencies pivot around 0%–2%. All other non-functional alleles are represented in the Caucasian population by less than 1% (Bradford, 2002; Zanger et al., 2004; Sistonen et al., 2007). Based on the genotype the activity score (AS) reflecting the metabolic capacity of 2D6 isoform can be calculated to classify the phenotype. In the metabolic phenotype of IMs, the genotype usually includes one non-functional allele and one allele with full or partial function, or two alleles with partial function (e.g., *4/*1, *5/*1, *3/*1). This leads to an AS within the following range: 0 < x < 1.25 (AS = 0.25–1 for IMs) (Bousman, et al., 2023).

The patient was identified to carry a heterozygous *4 allele genotype of the CYP2D6 gene. With the assumption that the other allele was functional, the patient’s phenotype was genetically predicted according to the AS range 0 < x < 1.25 as an IM with reduced enzyme activity. Less frequent non-functional alleles (Raimundo et al., 2000; Bousman, et al., 2023) were not tested and thus may have remained undetected. If they had been detected, the metabolic status would be poor metabolizer with deficient enzyme activity. Nevertheless, the probability of such phenotype category misclassification is considered minimal, as the prevalence of the untested non-functional alleles in Caucascian population is very rare.

### 2.9 Medical state at discharge and follow-up

The psychological examination that was performed between day 38 and day 45 revealed severe cognitive deficit with Mini-Mental State Examination score 23 out of 30 points. Moreover, impaired contact with reality and thought and perception disturbances were detected. Nevertheless, at the same time, delirium symptoms gradually faded. The patient started to cooperate; he was oriented in all qualities and started to take food and fluids. No delirium symptoms were observed at the discharge from the hospital on day 53. Medication at the time of discharge was olanzapine 15 mg/day, dabigatran etexilate 300 mg/day, metoprolol 25 mg/day, and tamsulosin hydrochloride 0.4 mg/day.

Thus, despite the high dose of paroxetine and the long persistence of toxic plasma levels, the patient recovered quite well. A psychological follow-up examination was recommended. The 18-month follow-up psychological examination revealed no cognitive deficit. At the three-year follow-up consultation via a telephone call to the psychiatrist, the patient experienced no chronic physical or psychic sequelae.

## 3 Discussion

We present a case of a patient intoxicated with paroxetine taken in an amount almost 20 times higher than the daily dose, in whom a toxic plasma level accompanied by delirium persisted over 1 month after the overdose. Phenoconversion from IM to PM is suggested to underlie the protracted intoxication in this patient, who, however, experienced no chronic sequelae.

This case highlights the importance of therapeutic drug monitoring, the value of knowledge of the dose, and the benefit of genetic testing focused on drug-metabolizing activity. All these aspects help predict the drug elimination time, though the phenotype is of the highest relevance. The t1/2 of paroxetine within therapeutic doses ranges between 12 and 44 h (Hiemke et al., 2018). In NMs, plasma t1/2 of paroxetine within less than 24 h was demonstrated after a single dose of 30 mg (Sindrup et al., 1992). A case study of an NM patient was reported in whom a very long t1/2 of paroxetine was measured (8.1 days) after an overdose of 2 g paroxetine and 1 g clorazepate (Hilleret et al., 2002). Contrary to that case, the IM patient reported here consumed the paroxetine alone and in half of that amount. Yet, we observed high plasma levels of paroxetine for weeks, and based on plasma level monitoring from the first available value, the t1/2 was approximately 9 days. We suppose that the chronic treatment with paroxetine could have contributed to the extremely long intoxication in this patient. It seems that at a steady-state condition of therapeutic doses of paroxetine, CYP2D6 is saturated and therefore insufficiently involved in elimination of the drug. This interpretation is in line with the conclusions of another study, in which phenoconversion to the PM phenotype was observed in NM genotyped patients who had undergone long-term therapy with paroxetine (Žourková and Hadašová, 2003), and of another reported case of paroxetine overdose (Hilleret et al., 2002). The reported patient was genotyped as an IM, i.e., with reduced metabolizing activity of the CYP2D6 enzyme. Furthermore, via the autoinhibition, phenoconversion to a PM with deficient enzyme activity likely occurred resulting in non-linear pharmacokinetics and therefore leading to the extremely long persistence of high plasma drug levels in a single overdose.

We cannot exclude the role and contribution of inflammation on elevated paroxetine concentration, as the CRP level was increased because of a urinary infection in the current patient at the time of admission. In inflammation and infection, CYP enzyme activity was previously shown to be down-regulated (Morgan, 2009) with a clinically relevant impact on the pharmacokinetics of CYP substrates (Lenoir et al., 2021). On the other hand, the inhibitory effects on the enzyme due to both the paroxetine overdose and preceding chronic therapy were likely already present at that time. Moreover, the initial levels of CRP were only moderately elevated, the infection was treated immediately after admission and resolved quickly with clear CRP decline. Therefore, we assume that the inflammatory response affected the biotransformation of paroxetine only minimally.

In addition, the impact of smoking should be considered since the patient was heavy smoker before his admission to the hospital. Smoking tobacco cigarettes induces the enzyme isoform CYP1A2, which generally persists for several days (Hiemke et al., 2018), leading to faster biotransformation of paroxetine. This effect, however, was not likely present since the patient discontinued smoking on admission due to his clinical status and long persisting high plasma levels of paroxetine were observed (Figure 2).

Interestingly, it has been shown that SSRIs are rarely fatal in overdose when taken alone (Barbey and Roose, 1998). Moderate overdoses of SSRIs (up to 30 times the standard daily dose) were associated with minor or no symptoms, while the intake of large amounts usually results in drowsiness, tremor, nausea, and vomiting. With very high doses of SSRIs (more than 75 times the common daily dose), seizures, ECG changes, and decreased consciousness may occur alone (Barbey and Roose, 1998). In one case of completed suicide with isolated intoxication with paroxetine, the post-mortem heart blood concentration of paroxetine was found to be 4,000 ng/mL (Vermeulen, 1998). In the current case, long-lasting delirium was observed that gradually faded when non-toxic level of paroxetine was reached after about 1 month. Therefore, we assume that delirium was most probably caused by protracted intoxication with paroxetine. Nevertheless, the suspected urinary infection, food and drink restriction, and possible dental infection could also have contributed to the delirium.

Currently, genetic testing is used to optimize medication therapy and CYP2D6-guided dosing recommendations for paroxetine are formulated, e.g., a lower paroxetine starting dose and slower titration may be considered for CYP2D6 IMs as compared to NMs due to autoinhibition of CYP2D6 and potential phenoconversion. In contrast, in ultrarapid metabolizers, fast biotransformation may lead to a lack of efficacy, and alternative drugs should be taken into account (Bousman, et al., 2023). Phenoconversion should be considered and addressed to ensure treatment efficacy and safety (Češková et al., 2022; den Uil et al., 2023; Hahn and Roll, 2023; Bousman, et al., 2023). Here, we demonstrated the importance of therapeutic drug monitoring and genotyping within the consideration of possible phenoconversion that might help explain the clinical status after drug intoxication. Specifically, drug plasma concentration monitoring, the patient’s individual clinical condition, pharmacotherapy history, genetic profile, and the dose are essential factors in the decision on sufficient monitoring time. In patients with a single overdose in the terrain of chronic administration of paroxetine, phenoconversion towards low CYP2D6 activity should also be considered in otherwise unexplained physical symptoms. In these cases, we therefore recommend, based on our current observations, longer than the standard 24-h monitoring of physical symptoms in the intensive care unit, including monitoring of plasma levels of paroxetine.

## Data Availability

The original contributions presented in the study are included in the article/supplementary material, further inquiries can be directed to the corresponding author.
